# Physical growth: is it a good indicator of development in early childhood in low- and middle-income countries?

**DOI:** 10.1186/s12887-019-1654-9

**Published:** 2019-08-08

**Authors:** Thach Duc Tran, Sara Holton, Hau Nguyen, Jane Fisher

**Affiliations:** School of Public Health and Preventive Medicine, Monash University, 553 St Kilda Road, Melbourne, VIC 3004 Australia

**Keywords:** Child growth, Early childhood development, Association, Low- and middle-income countries

## Abstract

**Background:**

Growth and early development (ECD) are vital outcomes for children. This study aimed to examine the association between child growth and overall development in children aged 3 to 5 years in low- and middle-income countries.

**Methods:**

A secondary analysis of nationally representative data collected in UNICEF’s Multiple Indicator Cluster Surveys (MICS) and national Demographic and Health Surveys (DHS). The early development of children aged 3 to 5 years from the randomly selected households was ascertained using a 10-item scale which assessed four developmental domains: language-cognitive, physical, socio-emotional, and approaches to learning with a total development score ranging from 0 (the least optimal) to 10 (the most optimal). Children’s growth, the height-for-age Z score (HAZ), was calculated using the WHO Child Growth Standards. Unadjusted (Pearson’s correlation coefficient, r) and adjusted estimations (standardised mean difference (SMD) adjusted for child sex, child age, and household wealth index) of the magnitude of the association between HAZ and ECD scores were calculated for each country.

**Results:**

Data contributed by 178,393 children aged 36 to 59 months from 55 countries were included in the analyses. The pooled r between HAZ and standardised ECD scores was 0.12 and the pooled adjusted SMD was 0.06. The r ranged from ~ 0 in Barbados, Lebanon, and Moldova to 0.32 in Pakistan and 0.36 in Nigeria. Overall, 47/55 countries had correlation coefficients less than the cut-off for a small association. The adjusted SMDs were ~ 0 in 20 countries. All SMDs were lower than the cut-off for a small effect size. The magnitudes of the association were highest in South Asia and lowest in Middle East and North Africa, and lowest in the highest HDI group.

**Conclusions:**

The association between growth and development in early childhood appears to be primarily a co-occurrence because the magnitude of the association varies among settings from no association in higher-income countries to a moderate level in low-income countries. In low-income countries, interventions targeting child growth and ECD should be integrated given their common risks frequency in these settings. Overall, growth is not a sensitive and therefore suitable indicator of child development.

**Electronic supplementary material:**

The online version of this article (10.1186/s12887-019-1654-9) contains supplementary material, which is available to authorized users.

## Background

Early childhood development (ECD) including cognitive, motor, and social-emotional domains is an important indicator that is positively associated with optimal adult health and productivity [1, 2]. Almost 43% of children under 5 years of age in countries classified as low- or middle-income in the World Bank Country Classification [3], for instance, India and Nepal, are at risk of failing to reach their developmental potential [4]. This figure was estimated based on the prevalence of children stunted and/or living in extreme poverty as a proxy indicator. ECD is increasingly recognised as an important World Developmental Indicator, including the Sustainable Development Goals [5].

There is a debate about the relationship between child linear growth and development. On one hand, some researchers argue that linear growth determines cognitive development in children. Since Porter’s study of 33,500 students in 1893, which, for the first time showed that “taller students performed better academically than did shorter students of the same age” [6], other studies drew similar conclusions [7, 8]. Although the data were cross-sectional, all interpreted the relationship as causal and concluded that a child’s body growth can influence their cognitive function. On the other hand, confounding can play a role in this relationship [9]. It is increasingly evident that common influences, e.g. the caregiving environment, may influence both of these outcomes.

The relationship between child linear growth and development is complicated. Physical growth can positively influence other developmental domains through the development of the brain and musculoskeletal system. Children with delays in cognitive, motor, and social-emotional development might have compromised interactions with caregivers and others. These can adversely affect the quality of care they receive and the opportunity to participate in activities that are crucial for healthy physical growth. Therefore, the relationship can be one way or the other way causal relationship or one confounded by common factors, or a combination of these.

In low- and middle-income countries child physical growth is often used as a proxy indicator of current early childhood development [1]. Child growth is relatively easy to assess as it does not involve arbitrary conceptualisation and operationalization and there are usually only minimal measurement errors [10, 11]. Child growth monitoring has been embedded in primary health care in almost every country [12]. Little is actually known about the strength of the association between child growth and ECD, and the consistency of this relationship among settings. If the association is strong and consistent, child growth failure can be used as an indicator of risk of developmental delay, and national policies for the integration of ECD interventions into child growth promotion programs to address both outcomes can be recommended for resource-constrained settings [13].

Several reviews about the association between child height for age and early childhood developmental outcomes have been published [14–16]. Among these, the meta-analysis by Sudfeld et al. [16] is the most recent and comprehensive. This analysis provided an estimation of the magnitude of the association between these two child outcomes. The correlation between child height for age and cognitive domain score in cross-sectional studies (11 studies) is 0.28 (95% CI, 0.19 to 0.36) and in prospective studies (5 studies) that child height for age is the predictor is 0.22 (95% CI, 0.17 to 0.27). The correlation coefficient between child height for age and motor domain score in cross-sectional studies (4 studies) is 0.24 (95% CI, 0.11 to 0.36) and in prospective studies (2 studies) is 0.29 (95% CI, 0.15 to 0.42). There was no estimation of the association between child growth and overall early childhood development. This meta-analysis included children aged from birth to 19 years. Subgroup analyses by child age indicated a significant difference in the magnitude of the association between child growth and cognitive development in children aged up to 2 years (0.24, 95% CI 0.14 to 0.33) and children aged > 2 years (0.09, 95% CI 0.05 to 0.13). As the number of studies included in the meta-analysis was relatively small (*n* = 11), no sensitivity analysis was conducted to confirm if the study setting influenced the magnitude of the association.

Two major multi-country studies, UNICEF’s Multiple Indicator Cluster Surveys (MICS) [17] and the Demographic and Health Surveys (DHS) [18], have collected data about child growth and early childhood development. These studies provide a unique cross-national and very large dataset that can be used to examine the association between child growth and ECD among a number of low- and middle-income countries.

The aim of this study was to estimate the magnitude of the association between child growth and overall development (ECD) among children aged 3 to 5 years in diverse low and middle-income settings. In particular, the study aimed to determine if the association was consistent across countries, geographic regions, and the Human Development Index groups.

## Methods

### Study design

This is a secondary analysis of data collected from the Multiple Indicator Cluster and the Demographic and Health Surveys.

The MICS are household surveys about women’s and children’s health initiated by the United Nations Children’s Fund (UNICEF) and implemented in up to five rounds in 112 low- and middle-income countries over the last 22 years. Since the fourth round (2010–2012), the early development of children aged 3 to 5 years has been assessed along with their anthropometric information.

The DHS are also household surveys that collect data about women’s and children’s health. More than 300 surveys have been conducted in over 90 low- and middle-income countries in the last 35 years. Assessment of children’s early childhood development has been included in the DHS since 2010.

### Sample

The DHS and MICS used similar multistage, cluster-sampling methods to recruit a large nationally representative household sample for each survey in each country. Every child aged 3 to 5 years in the selected household was evaluated.

This study included only data from the surveys which collected information about early childhood development and children’s anthropometric data. If a country had participated in more than one round of the survey (both DHS and MICS), only data from the most recent survey was included.

### Data sources

Overall early childhood development (ECD) was assessed in DHS and MICS using the same scale which was developed by an expert group at UNICEF. The scale includes 10 yes/no items on four developmental domains: language-cognitive, physical, socio-emotional, and approaches to learning [19]. The items were derived from a broad set of UNICEF’s early childhood development indicators and pilot-tested in Jordan, the Philippines and Kenya.

Each item is scored 1 if the child can achieve the task and 0 if they are not able to. The scale yields a total early childhood development score ranging from 0 (the least optimal development) to 10 (the most optimal development).

Child height was measured directly using UNICEF’s standardised equipment and techniques by trained interviewers during home visits [20]. Each child’s height-for-age Z score (HAZ) was calculated using the WHO Child Growth Standards (WHO Anthro version 3.2.2) [21] and based on their height, age, and sex.

Household socioeconomic status was assessed using information about household characteristics including the main materials used to construct the household dwelling’s wall, roof and floor; main type of fuel used for cooking; source of drinking water; type of sanitation facility; and 12 durable household assets. A household wealth index was constructed for the DHS/MICS using the World Bank’s techniques [22].

The United Nations Development Program’s national Human Development Index (HDI) is a proxy indicator of the social and economic status of a country that includes long and healthy life, education, and the standard of living. The country’s HDI the year survey data were collected was obtained from the UNDP’s Human Development Reports. The HDI ranges from 0 (lowest) to 1 (highest) and is classified into very high (≥0.800), high (0.700 to 0.799), medium (0.550 to 0.699), and low categories (0.550 or less) [23].

### Analysis

Data were analysed using Stata Release 14 [24]. All analyses used SVY commands that are designed to analyse complex survey data and to adjust the estimations of all statistics calculated for survey sampling design characteristics [25]. In every DHS/MICS survey, the cluster codes and cluster sampling weights (that are equal/proportional to the inverse of the probability being sampled) were provided in the data sets and were used to control for those.

Unadjusted and adjusted estimations and 95% CI of the magnitude of the association between HAZ and ECD scores were calculated at the individual level for each country. The unadjusted estimation is the Pearson’s correlation coefficient (r) of HAZ and ECD scores: |r| ≥ 0.50 is considered large, ≥ 0.30 to 0.49 medium, and ≥ 0.10 to 0.29 small [26]. The adjusted estimation is the adjusted standardised mean difference (SMD) calculated using a multiple linear regression predicting standardised ECD scores from HAZ taking into account child sex, child age, and household wealth index. Household wealth was used as a proxy variable for all potential confounders. SMD is interpreted as the number of standard deviations in ECD scores that change for each unit increase in HAZ. |SMD| ≥ 0.80 is considered large, ≥ 0.50 to 0.79 medium, and ≥ 0.20 to 0.49 small [26].

The pooled estimate for all countries was calculated as the median of all country estimations (the country level). Similarly, the pooled estimations at the country level for groups of countries on the basis of several characteristics including region and HDI group were calculated. At the country level, the association between mean HAZ and mean ECD score was calculated using Spearman correlation.

Only children with complete early childhood development data were included in the analyses.

## Results

Data contributed by 178,393 children aged 36 to 59 months from 55 countries were included in the analyses (Additional file 1: Table S1). The 55 countries were in low (20 countries), medium (23), and high (22) HDI groups. There were 5 countries from East Asia, 21 from Sub-Saharan Africa, 9 from Europe and Central Asia, 10 from Latin America and Caribbean, 6 from Middle East and North Africa region and 4 from South Asia.

The country mean HAZ and ECD scores are presented in Table 1. The mean HAZ ranged from − 2.36 in Burundi to 0.48 in Barbados and Serbia. The mean ECD scores ranged from 4.35 in Burundi to more than 8.0 in Trinidad and Tobago, and Barbados.

**Table 1:** Means of HAZ and ECD scores and association between those by country

Country	Mean HAZ^(a)^	Mean ECD^(b)^	Correlation^(c)^ [95% CI]	SMD^(d)^ [95% CI]
Algeria	− 0.70	5.90	**0.05 [0.02;0.08]**	0.01 [− 0.02;0.04]
Bangladesh	− 1.90	5.67	**0.21 [0.19;0.23]**	**0.14 [0.11;0.16]**
Barbados	0.48	8.44	−0.03 [− 0.18;0.12]	− 0.02 [− 0.15;0.10]
Belize	− 0.93	7.24	**0.15 [0.08;0.21]**	0.05 [− 0.01;0.10]
Benin	− 1.59	5.31	**0.18 [0.15;0.21]**	**0.07 [0.04;0.10]**
Bhutan	−1.72	5.99	**0.11 [0.07;0.15]**	**0.05 [0.01;0.10]**
Bosnia and Herzegovina	0.35	7.40	**0.06 [−0.00;0.12]**	**0.06 [0.02;0.11]**
Burundi	− 2.36	4.35	**0.22 [0.18;0.26]**	**0.12 [0.08;0.17]**
Cambodia	−1.70	5.98	**0.14 [0.09;0.19]**	**0.11 [0.06;0.16]**
Cameroon	−1.36	5.56	**0.23 [0.20;0.27]**	**0.09 [0.06;0.11]**
Central African Republic	−1.96	4.74	**0.11 [0.08;0.15]**	**0.07 [0.04;0.10]**
Chad	−1.62	4.44	**0.11 [0.08;0.14]**	**0.05 [0.02;0.07]**
Congo	−1.30	4.80	**0.10 [0.05;0.15]**	**0.06 [0.00;0.12]**
Democratic Republic of Congo	−2.05	4.81	**0.15 [0.12;0.18]**	**0.07 [0.04;0.10]**
El Salvador	−0.89	6.41	**0.15 [0.11;0.18]**	**0.08 [0.03;0.12]**
Ghana	−1.43	6.00	**0.20 [0.17;0.24]**	**0.13 [0.08;0.18]**
Guinea Bissau	−1.38	5.12	**0.14 [0.10;0.18]**	**0.07 [0.03;0.11]**
Guyana	−0.55	7.43	**0.20 [0.15;0.26]**	**0.09 [0.04;0.14]**
Honduras	−1.36	5.98	**0.15 [0.11;0.18]**	0.02 [−0.02;0.06]
Iraq	−1.01	5.66	**0.05 [0.03;0.07]**	**0.03 [0.01;0.06]**
Jordan	−0.57	5.52	**0.04 [0.00;0.08]**	0.01 [− 0.07;0.09]
Kazakhstan	−0.15	6.80	**0.05 [0.01;0.10]**	0.02 [− 0.02;0.06]
Kenya	−1.47	5.64	**0.16 [0.13;0.20]**	**0.08 [0.06;0.11**]
Kosovo	−0.29	6.68	0.07 [−0.01;0.15]	0.01 [− 0.06;0.09]
Kyrgyzstan	−1.02	5.79	**0.06 [0.01;0.11]**	0.03 [− 0.02;0.09]
Lao	−2.18	6.38	**0.16 [0.13;0.19]**	**0.07 [0.04;0.09]**
Lebanon	−0.79	6.72	−0.02 [− 0.09;0.06]	0.01 [− 0.06;0.07]
Macedonia	− 0.07	7.56	**0.15 [0.06;0.23]**	**0.13 [0.04;0.22]**
Malawi	−1.79	5.30	**0.09 [0.07;0.12]**	**0.04 [0.01;0.06]**
Mali	−1.42	5.24	**0.10 [0.07;0.12]**	**0.04 [0.02;0.07]**
Mauritania	−1.29	5.51	**0.16 [0.13;0.19]**	**0.08 [0.06;0.11]**
Mexico	−0.81	6.55	**0.08 [0.05;0.12]**	**0.04 [−0.01;0.10]**
Moldova	−0.37	6.88	0.01 [−0.07;0.09]	−0.05 [− 0.13;0.03]
Mongolia	− 0.89	6.29	**0.05 [0.01;0.09]**	**0.01 [−0.04;0.06]**
Montenegro	0.37	7.44	**0.12 [0.04;0.20]**	**0.05 [−0.02;0.13]**
Nepal	−2.01	5.56	**0.24 [0.20;0.28]**	**0.11 [0.06;0.15]**
Nigeria	−1.78	5.69	**0.36 [0.35;0.38]**	**0.10 [0.08;0.13]**
Pakistan	−1.88	5.64	**0.32 [0.30;0.33]**	**0.16 [0.15;0.17]**
Palestine	−0.46	5.87	**0.10 [0.07;0.14]**	**0.06 [0.03;0.10]**
Paraguay	−0.32	6.42	**0.15 [0.11;0.20]**	**0.04 [−0.03;0.10]**
Rwanda	−1.71	5.54	**0.17 [0.12;0.23]**	**0.06 [0.01;0.12]**
Sao Tome and Principe	−0.92	5.43	**0.17 [0.10;0.24]**	**0.11 [0.03;0.18]**
Serbia	0.48	7.70	**0.16 [0.10;0.22]**	**0.07 [0.01;0.14]**
Sierra Leone	−2.12	4.64	**0.12 [0.08;0.15]**	**0.06 [0.03;0.09]**
St Lucia	0.09	7.85	0.05 [−0.15;0.24]	−0.04 [− 0.20;0.13]
Suriname	− 0.62	6.00	**0.16 [0.09;0.22]**	0.04 [−0.02;0.11]
Swaziland	−1.26	5.63	**0.17 [0.11;0.23]**	**0.08 [0.02;0.14]**
Thailand	−0.61	7.81	**0.05 [0.01;0.08]**	0.01 [−0.03;0.05]
Timor-Leste	−1.85	5.33	**0.08 [0.01;0.15]**	0.01 [−0.04;0.07]
Togo	−1.69	4.85	**0.19 [0.15;0.24]**	**0.06 [0.01;0.11]**
Trinidad and Tobago	0.06	8.16	0.04 [−0.06;0.13]	0.02 [−0.05;0.08]
Tunisia	−0.40	6.03	0.04 [−0.02;0.10]	0.00 [−0.06;0.06]
Turkmenistan	−0.67	6.85	**0.09 [0.04;0.14]**	**0.07 [0.02;0.12]**
Uganda	−1.31	5.51	**0.14 [0.09;0.19]**	**0.06 [0.02;0.10]**
Zimbabwe	−1.35	5.41	**0.12 [0.09;0.15]**	**0.06 [0.03;0.09]**
Median	−1.26	5.87	0.12	0.06

^(a)^Height-for-age Z score: calculated using the WHO Anthro Version 3.2.2 software (2011)

^(b)^Early childhood development index

^(c)^Pearson correlation coefficient between HAZ and standardised ECD score

^(d)^Standardised mean difference: calculated using a multiple linear regression predicting standardised ECD scores from HAZ taking into account child sex, child age, and household wealth index

Bolded correlation coefficient/SMD: statistically significant

Overall, the pooled correlation coefficients between HAZ and standardised ECD scores was 0.12 and the pooled adjusted SMD was 0.06 (Table 1). In 7 countries including Barbados, Kosovo, Lebanon, Moldova, St Lucia, Trinidad and Tobago, Tunisia, the correlation coefficients were not statistically significantly different to zero. The highest correlation coefficients were in Pakistan (0.32) and Nigeria (0.36). Overall, 47/55 countries had correlation coefficients less than the cut-off for a small association. The adjusted SMD were not statistically significantly different to zero in 20 countries. All SMD were lower than the cut-off for a small effect size.

Differences between the mean HAZ and mean ECD scores were found among HDI groups (Table 2). The higher the HDI group, the higher the mean HAZ and mean ECD scores. The median of the correlation coefficients and adjusted SMD were slightly different between low and medium HDI groups and higher than those in the high HDI group which were close to 0. When adjusted for child age, child sex and household wealth, the magnitudes of the association became smaller in all HDI groups.

**Table 2:** Medians of country means of HAZ and ECD scores and association between those by HDI groups and regions

	Median mean HAZ^(a)^	Median mean ECD^(b)^	Median correlation^(c)^	Median SMD^(d)^
Human development index group				
Low	− 1.62	5.31	0.16	0.07
Medium	−1.02	5.98	0.14	0.06
High	−0.40	6.80	0.05	0.01
Region				
East Asia and the Pacific	−1.70	6.29	0.08	0.01
Sub-Sahara Africa	−1.47	5.31	0.16	0.07
Europe and Central Asia	−0.15	6.88	0.07	0.05
Latin America and Caribbean	−0.58	6.90	0.15	0.04
Middle East and North Africa	−0.64	5.88	0.04	0.01
South Asia	−1.89	5.66	0.22	0.13

^(a)^Height-for-age Z score: calculated using the WHO Anthro Version 3.2.2 software (2011)

^(b)^Early childhood development index

^(c)^Pearson correlation coefficient between HAZ and standardised ECD score

^(d)^Standardised mean difference: calculated using a multiple linear regression predicting standardised ECD scores from HAZ taking into account child sex, child age, and household wealth index

There were differences among regions regarding HAZ and ECD scores and the magnitude of the association between those outcomes (Table 2). Mean HAZ was lowest in South Asian and East Asian and Pacific countries. Mean ECD scores were lowest in Sub-Saharan African countries. The magnitude of the association between HAZ and ECD scores was highest in South Asia and lowest in Middle East and North Africa.

**Fig. 1:**
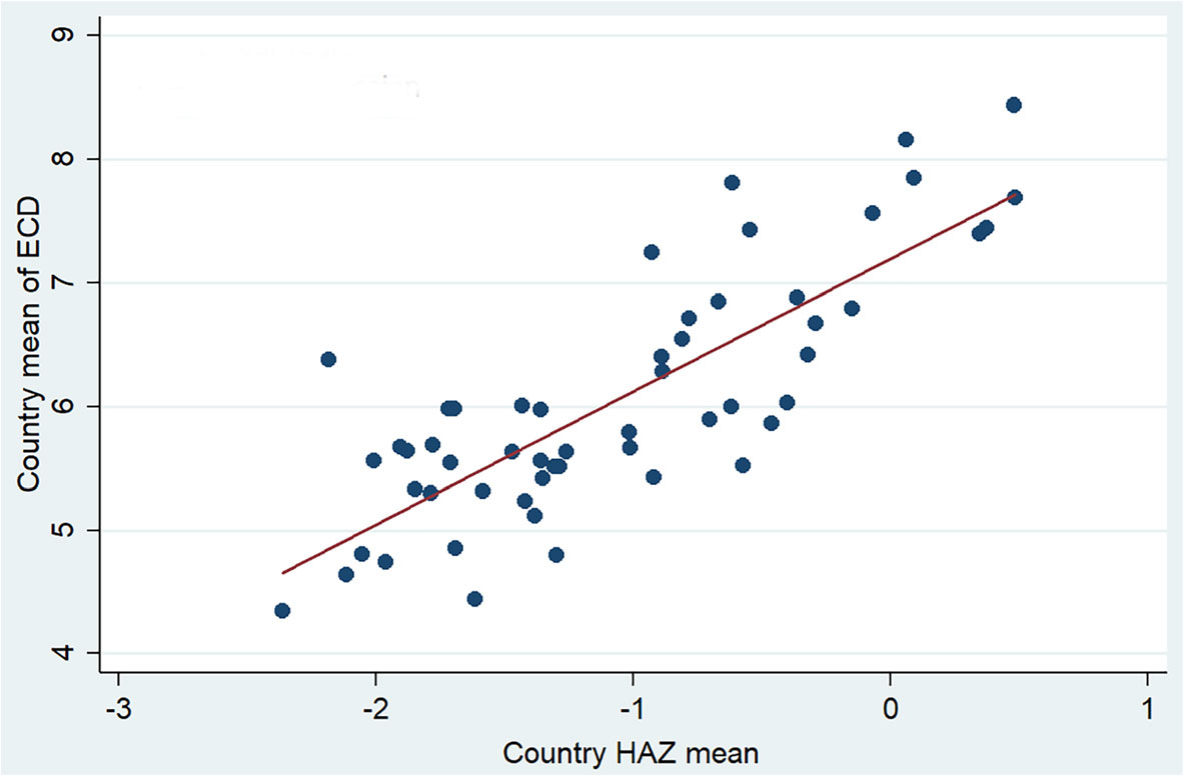
Plots of country means of height-for-age Z score (HAZ) and Early Childhood Development score (ECD). Red line: Linear regression

There was a strong positive correlation between mean HAZ and mean ECD scores at the country level (Fig. 1). Spearman correlation coefficient (rho) was 0.76 (*p* < 0.001). Both mean HAZ and mean ECD scores were highly associated with HDI (rho = 0.83 and 0.86, respectively).

## Discussion

This is a unique study examining the association between child growth and early childhood development in a large number of low and lower-middle income settings. Overall, the magnitude of the correlation between these child outcomes was low. The association varied significantly among countries and reduced significantly when controlled for child age, sex, and household wealth. Child growth and early childhood development indices were strongly associated at the country level.

The association between two variables can be due to a causal relationship or the result of a co-occurrence due to common risk factors, or a combination of these. Evidence from longitudinal studies suggests a negative effect of growth retardation on child cognitive and motor development [27–29]. Nevertheless, such studies have used an observational design that is not able to control for all potential confounders and therefore, it is not possible to conclude that a causal relationship exists between child growth and ECD. Accordingly, there is currently no rigorous evidence which indicates that child development affects child growth or that child growth affects child development. Child growth and development share a number of common determinants [30, 31] including insufficient access to nutrition, exposure to infectious diseases, and an adverse ‘care environment’ including a lack of responsive and sensitive care and resources at both the household and community level.

These data indicate that the association between child growth and development is mainly a co-occurrence. The correlation coefficients varied from no association to a moderate association across countries. The association also varied among HDI groups and regions with higher magnitudes of the association apparent in more disadvantaged settings. After adjusting for household wealth, the magnitude of the association reduced significantly and became zero in more than one third of the countries included in the analyses. These findings indicate that the magnitude of the association is mostly explained by variance in the common risk factors, in particular, the care environment (e.g. lack of resources and insensitivity care). In South Asian and Sub-Sahara African countries where the care environment is often poor, the magnitude of the association was higher. In high HDI countries, where the care environments are better, there was a modest association between the outcomes. These findings suggest that when extreme adverse care environmental risks are absent, physical growth and ECD are affected mainly by its determinants. The high correlation between child growth and development indices at a country level does not imply that there is a high correlation between those outcomes at the individual level. That both country child growth and development indices are highly positively associated with HDI suggests that HDI plays a key role in the high association between these child outcomes at the country level. Therefore, at the country level, HDI or child growth index can be used to predict the ECD index in low- and middle-income countries.

This study did not include data from high-income countries. In the past, studies in these settings have suggested a significant association between child linear growth and child cognitive ability [6–8, 32]. However, the effect size of the associations has not been analysed systematically using nationally representative data for each country. The socio-economic characteristics of high-income countries are substantially different from those of low- and middle-income countries and lead to substantial differences in the caregiving environment for children. Therefore, the question of whether child growth can predict the ECD index in high-income settings remains.

The pooled correlation coefficient between child growth and development in this study is about half of that found in Sudfeld et al.’s meta-analysis. The number of settings included in the meta-analysis is much smaller than in this study (11 verse 55 countries). Similar to our study, the magnitudes of the correlation in the studies included in the meta-analysis varied from 0.05 in China to 0.33 in Ethiopia. The I-squared statistic which describes the percentage of variation across studies that is due to heterogeneity rather than chance, was 84% for the motor domain and 92% for the cognitive domain indicating considerable heterogeneity in the results of the studies included in Sudfield et al.’s meta-analysis. Our study demonstrates that the relationship between child growth and ECD varies by the human development status of the country.

We use height-for-age as the index of child growth in this study. There are several other common indicators of child growth that can be used including weight-for-age, weight-for-height, body mass index (weight in kg divided by height in metres squared), mid-upper arm circumference, and head circumference. Height-for-age is regarded as the best child growth indicator because it reflects cumulative linear growth and can be measured accurately using a widely available tool and a standardised method [10]. Other methods that are related to weight can be confounded by short-term problems like starvation or severe disease. We have repeated the analyses using weight-for-age and weight-for-height z-scores as the indicator of child growth. The results were relatively similar to the results of height-for-age z-scores (Please contact the authors for the results). Therefore, we are confident that the main findings of this study would not change if we used another indicator of child growth.

We acknowledge some limitations of this study. This was a secondary analysis of existing survey data so it was not possible to alter the data collection design. Firstly, the tool used to assess ECD in MICS and DHS is a brief and parent-self-reported scale which does not involve assessor observation of the child. A locally-validated, abilities, child-direct-assessment like the Bayley Scales of Infant and Toddler Development – Third Edition [33] produces more comprehensive and objective indices of ECD. However, direct assessments of individual children require considerable resources and are not feasible for use in large-scale surveys. The scale used in the MICS and DHS was rigorously developed by UNICEF’s expert team and tested in multiple low- and middle-income settings [19]. Second, the MICS and DHS did not collect a comprehensive set of common risk factors for child growth and development. We used household wealth as a proxy indicator of the home environment and HDI as a proxy indicator of the country environment which does not include country-specific policies about access to early childhood education and health care. Finally, this study included only children aged 3 to 5 years. Child growth and overall development are cumulative over time and cannot be changed in a short period. Therefore, the outcomes measured at 3 to 5 years can reflect the status and changes in the first two years of life but are not a direct measure of early growth and development.

The results of this study suggest that child growth is not suitable for use as an indicator of child development. Therefore, there is an urgent need for an age-appropriate scale which assesses early childhood development and that can be used in primary health care in low- and middle-income countries. Such a scale should be more detailed than the scale used in this study to evaluate every domain of early childhood development including cognitive, motor, social and emotional behaviour. Our findings indicate that ECD interventions should not focus predominantly on child growth and, in low-income countries, interventions targeting child growth and ECD should be integrated as there are common risk factors for both of these and they are widespread in these settings.

## Conclusions

The association between growth and development in early childhood appears to be a co-occurrence. The magnitude of the association varies across settings from no association to a moderate level that appears to depend on the presence of common factors such as adverse home care environment and country-level human development indicators. Future studies should verify this finding in children of other age groups and in high-income settings.

## Additional file

Additional file 1: **Table S1.** Characteristics by country. (DOCX 20 kb)

## Data Availability

Data are available for public use at http://mics.unicef.org/surveys and https://dhsprogram.com/data/available-datasets.cfm.

## Abbreviations

DHS: Demographic and Health Surveys
ECD: Early childhood development
HAZ: Height-for-age Z score
HDI: Human Development Index
MICS: Multiple Indicator Cluster Surveys
