# Supplementary material for: Physical growth: is it a good indicator of development in early childhood in low- and middle-income countries?
Source: BMC Pediatr. 2019 Aug 8;19:276. doi: 10.1186/s12887-019-1654-9 (PMC6686501; doi:10.1186/s12887-019-1654-9)
Supplement: Supplementary file 1 — Table S1. Characteristics by country. (DOCX 20 kb) [file 12887_2019_1654_MOESM1_ESM.docx]

Additional file 1: table 1: Characteristics by country

| Country | Data source | Region | Year | HID group | sample | Girl  (%) | Child  Age (months) |
| --- | --- | --- | --- | --- | --- | --- | --- |
| Algeria | MICS | Middle East and North Africa | 2012 | 3 | 5558 | 47.8 | 47.7 |
| Bangladesh | MICS | South Asia | 2012 | 2 | 8791 | 48.0 | 48.0 |
| Barbados | MICS | Latin America and Caribbean | 2012 | 3 | 202 | 44.6 | 49.3 |
| Belize | MICS | Latin America and Caribbean | 2015 | 3 | 1092 | 48.6 | 47.8 |
| Benin | MICS | West and Central Africa | 2014 | 1 | 4818 | 50.7 | 47.5 |
| Bhutan | MICS | South Asia | 2010 | 2 | 2420 | 49.0 | 46.7 |
| Bosnia and Herzegovina | MICS | Europe and Central Asia | 2011 | 3 | 1031 | 51.5 | 47.4 |
| Burundi | DHS | Eastern and Southern Africa | 2016 | 1 | 2240 | 49.9 | 48.4 |
| Cambodia | DHS | East Asia and the Pacific | 2014 | 2 | 1603 | 49.7 | 47.7 |
| Cameroon | MICS | West and Central Africa | 2014 | 1 | 2812 | 50.7 | 46.8 |
| Central African Republic | MICS | West and Central Africa | 2010 | 1 | 3771 | 51.9 | 46.1 |
| Chad | MICS | West and Central Africa | 2010 | 1 | 7063 | 50.3 | 48.0 |
| Congo | DHS | West and Central Africa | 2011 | 2 | 1483 | 50.0 | 47.1 |
| Democratic Republic of Congo | MICS | West and Central Africa | 2010 | 1 | 4045 | 50.0 | 46.2 |
| El Salvador | MICS | Latin America and Caribbean | 2014 | 2 | 2987 | 49.3 | 48.1 |
| Ghana | MICS | West and Central Africa | 2011 | 2 | 3236 | 49.5 | 47.6 |
| Guinea Bissau | MICS | West and Central Africa | 2014 | 1 | 2939 | 50.4 | 47.1 |
| Guyana | MICS | Latin America and Caribbean | 2014 | 2 | 1329 | 48.2 | 48.2 |
| Honduras | DHS | Latin America and Caribbean | 2012 | 2 | 2786 | 47.4 | 47.7 |
| Iraq | MICS | Middle East and North Africa | 2011 | 2 | 13480 | 49.3 | 47.5 |
| Jordan | DHS | Middle East and North Africa | 2012 | 3 | 2610 | 49.5 | 47.5 |
| Kazakhstan | MICS | Europe and Central Asia | 2015 | 3 | 2243 | 49.3 | 47.8 |
| Kenya | MICS | Eastern and Southern Africa | 2013 | 1 | 3421 | 49.1 | 49.2 |
| Kosovo | MICS | Europe and Central Asia | 2013 | 3 | 660 | 47.3 | 48.1 |
| Kyrgyzstan | MICS | Europe and Central Asia | 2014 | 2 | 1783 | 48.7 | 47.5 |
| Lao | MICS | East Asia and the Pacific | 2011 | 2 | 4474 | 48.4 | 47.7 |
| Lebanon | MICS | Middle East and North Africa | 2011 | 3 | 689 | 46.7 | 47.9 |
| Macedonia | MICS | Europe and Central Asia | 2011 | 3 | 558 | 50.0 | 48.6 |
| Malawi | MICS | Eastern and Southern Africa | 2013 | 1 | 7707 | 50.1 | 47.4 |
| Mali | MICS | West and Central Africa | 2015 | 1 | 6415 | 48.4 | 46.5 |
| Mauritania | MICS | West and Central Africa | 2015 | 1 | 4389 | 50.6 | 47.0 |
| Mexico | MICS | Latin America and Caribbean | 2015 | 3 | 3328 | 49.0 | 48.1 |
| Moldova | MICS | Europe and Central Asia | 2012 | 2 | 733 | 46.7 | 47.4 |
| Mongolia | MICS | East Asia and the Pacific | 2013 | 3 | 2970 | 49.5 | 47.9 |
| Montenegro | MICS | Europe and Central Asia | 2013 | 2 | 645 | 46.4 | 47.9 |
| Nepal | MICS | South Asia | 2014 | 2 | 2261 | 48.1 | 47.9 |
| Nigeria | MICS | West and Central Africa | 2016 | 1 | 11529 | 49.3 | 47.3 |
| Pakistan | MICS | South Asia | 2014 | 1 | 18313 | 49.0 | 47.3 |
| Palestine | MICS | Middle East and North Africa | 2014 | 2 | 3220 | 48.2 | 47.6 |
| Paraguay | MICS | Latin America and Caribbean | 2016 | 2 | 1835 | 48.1 | 48.0 |
| Rwanda | DHS | Eastern and Southern Africa | 2015 | 1 | 1301 | 48.4 | 46.8 |
| Sao Tome and Principe | MICS | West and Central Africa | 2014 | 2 | 851 | 49.9 | 47.9 |
| Serbia | MICS | Europe and Central Asia | 2014 | 3 | 1191 | 49.4 | 48.1 |
| Sierra Leone | MICS | West and Central Africa | 2010 | 1 | 3675 | 49.7 | 47.2 |
| St Lucia | MICS | Latin America and Caribbean | 2012 | 3 | 122 | 50.8 | 48.1 |
| Suriname | MICS | Latin America and Caribbean | 2010 | 3 | 1283 | 51.3 | 47.7 |
| Swaziland | MICS | Eastern and Southern Africa | 2014 | 1 | 1073 | 48.9 | 47.6 |
| Thailand | MICS | East Asia and the Pacific | 2012 | 3 | 4061 | 49.9 | 47.8 |
| Timor-Leste | DHS | East Asia and the Pacific | 2016 | 2 | 865 | 48.6 | 42.7 |
| Togo | MICS | West and Central Africa | 2010 | 1 | 1803 | 48.8 | 47.1 |
| Trinidad and Tobago | MICS | Latin America and Caribbean | 2011 | 3 | 527 | 51.8 | 47.2 |
| Tunisia | MICS | Middle East and North Africa | 2011 | 3 | 1162 | 46.0 | 47.3 |
| Turkmenistan | MICS | Europe and Central Asia | 2015 | 2 | 1495 | 47.6 | 47.6 |
| Uganda | DHS | Eastern and Southern Africa | 2016 | 1 | 1609 | 50.4 | 47.6 |
| Zimbabwe | MICS | Eastern and Southern Africa | 2014 | 1 | 3906 | 50.5 | 47.2 |
